# The Effectiveness of Cognitive Behavioral Therapy With Mindfulness and an Internet Intervention for Obesity: A Case Series

**DOI:** 10.3389/fnut.2018.00056

**Published:** 2018-06-27

**Authors:** Keizaburo Ogata, Ken I. Koyama, Marie Amitani, Haruka Amitani, Akihiro Asakawa, Akio Inui

**Affiliations:** ^1^Department of Clinical Psychology, Kitasato University East Hospital, Sagamihara, Japan; ^2^Department of Psychosomatic Internal Medicine, Kagoshima University Graduate School of Medical and Dental Sciences, Kagoshima, Japan; ^3^Faculty of Integrated Human Studies and Social Sciences, Fukuoka Prefectural University Graduate School of Human and Social Sciences, Fukuoka, Japan; ^4^Education Center for Doctors in Remote Islands and Rural Areas, Kagoshima University Graduate School of Medical and Dental Sciences, Kagoshima, Japan

**Keywords:** obesity, overweight, cognitive behavioral therapy, mindfulness, dropout prevention, weight maintenance

## Abstract

It is difficult for obese (body mass index of more than 30) and overweight (body mass index of 25–30) people to reduce and maintain their weight. The aim of this case series was to examine the effectiveness of a new cognitive behavioral therapy (CBT) program that combines mindfulness exercises (e.g., the raisin exercise and breathing exercises) and an online intervention to prevent dropout and subsequent weight gain in overweight participants. This case series included three participants, for whom previous weight reduction programs had been unsuccessful. All participants completed the program (60-min, group sessions provided weekly for 9 weeks) and an 18-month follow-up assessment. Results showed that all participants succeeded in losing weight (loss ranged from 5.30 to 8.88% of their total body weight). Although rebound weight gain is commonly observed in the first year following initial weight loss, the follow-up assessment showed that participants achieved further weight loss during the 18-month follow-up period. These results suggest that a CBT program that comprises mindfulness and an online intervention may be an effective method for weight loss and maintenance, and may prevent dropout in obese and overweight individuals.

Trial Registration: This case series was registered at www.umin.ac.jp with identifier UMIN000029664.

## Introduction and background

The basic strategies involved in the treatment of obesity (body mass index [BMI] over 30) and overweight (BMI between 25 and 30) include lifestyle improvements, exercise therapy, and cognitive behavioral therapy (CBT). The structure of standard CBT for obesity and overweight includes self-monitoring, goal-setting, stimulus control, behavioral substitution, and cognitive restructuring (1). Numerous studies have reported that the use of CBT is successful in reducing patients' weight during treatment. However, many patients subsequently regain weight and fail to maintain weight reduction following the completion of treatment (2). In addition, the results of a randomized controlled trial by Cooper et al. (3) found that a new treatment program aimed at weight maintenance was no more effective, relative to basic treatment, in preventing patients from regaining weight (3). These findings indicate that treatment for obesity should be extended to maintain patients' weight loss.

Moreover, patients frequently drop out of obesity treatment programs, which further complicates the success of treatment (4, 5). Although increasing the frequency of the interventions could prevent dropout and maintain patients' motivation, it could also lead to several problems involving health resources by increasing patients' physical, temporal, and psychological burden, which could result in further dropout. Therefore, interventions that are implemented frequently and that reduce these types of burden are required. In this regard, the provision of CBT via the Internet (iCBT) has recently been shown to be as effective as face-to-face treatment for conditions such as depression, anxiety, and insomnia (6–8). Moreover, therapist-guided iCBT may be less time consuming, relative to face-to-face treatment, for both individual and group therapy (9). In addition, mindfulness, which is one of the main concepts in third-wave CBT, is well known for its effectiveness in the treatment of anxiety, depression, and impulsive eating (10–12). Given the promise of both iCBT and mindfulness interventions, we developed a new program involving mindfulness exercises and an online intervention for obesity to prevent participant dropout and improve psychological health. The purpose of this case series was to determine the effectiveness of Cognitive Behavioral Therapy with Mindfulness Exercise and an Internet intervention for Obesity (CBT-MIO).

## Method

### Participants

Participants were recruited via an advertisement placed on the notice board at Kagoshima University in December 2014. The inclusion criteria were as follows: (a) between 18 and 60 years of age, (b) BMI (weight in kg/height in m^2^) between 25.0 and 40.0, (c) previous lack of success in weight reduction programs, (d) availability to participate in treatment for 9 weeks, (e) access to the Internet on a daily basis, and (f) willingness to participate in the study. The exclusion criteria were as follows: (a) consultation with a doctor for type 1 or 2 diabetes, cardiovascular disease, or mental health disorders, (b) pregnancy, and (c) weight loss within the preceding 6 months because of weight loss success. Table 1 shows the characteristics of participants. Three participants (two female) were included in the study. The age of participants was in 20–30 years, and they were defined as overweight. One of Trait anxiety was high (Participant B), however State anxiety of all participants was in the normal range (13, 14). All participants attended a follow-up assessment 18 months after the final weight loss session.

**Table 1:** Participant characteristics.

	**Participant A**	**Participant B**	**Participant C**
Age	20–25	20–25	25–30
Height (m)	1.55	1.71	1.51
Weight (kg)	60.80	86.00	57.50
BMI (kg/m^2^)	25.31	29.41	25.22
**STAI**			
State	37	34	31
Trait	39	46	33
**DEBQ**			
Restrained	3.10	1.41	3.80
Emotional	3.69	1.31	1.54
External	4.10	4.10	2.30
**FFMQ**			
Observing	26	18	25
Describing	31	24	24
Acting with awareness	28	29	27
Nonjudging	21	27	30
Nonreactivity	18	19	23

*BMI, Body Mass Index; STAI, State-Trait Anxiety Inventory; DEBQ, Dutch Eating Behavior Questionnaire; FFMQ, Five Facet Mindfulness Questionnaire*.

### CBT-MIO

CBT-MIO was developed based on a well-known CBT program for weight loss and maintenance (2, 3). CBT-MIO aims to aid participants in losing between 5% to 10% of their total body weight and consists of four elements: (a) a psychoeducational intervention designed to promote a healthy diet and physical exercise, and to reduce self-sabotaging thoughts; (b) self-monitoring of daily food-intake using a notebook and the use of a social networking sites (SNSs) to upload photographs of self-reported food consumption during the final 4 weeks of the program; (c) mindfulness exercises [e.g., raisin exercise and mindful breathing; (15)] to increase distress tolerance, improve healthy coping strategies, and reduce maladaptive coping strategies (e.g., avoidant and impulsive coping styles that involve emotional eating); and (d) relearning adaptive eating habits.

**Figure 1:**
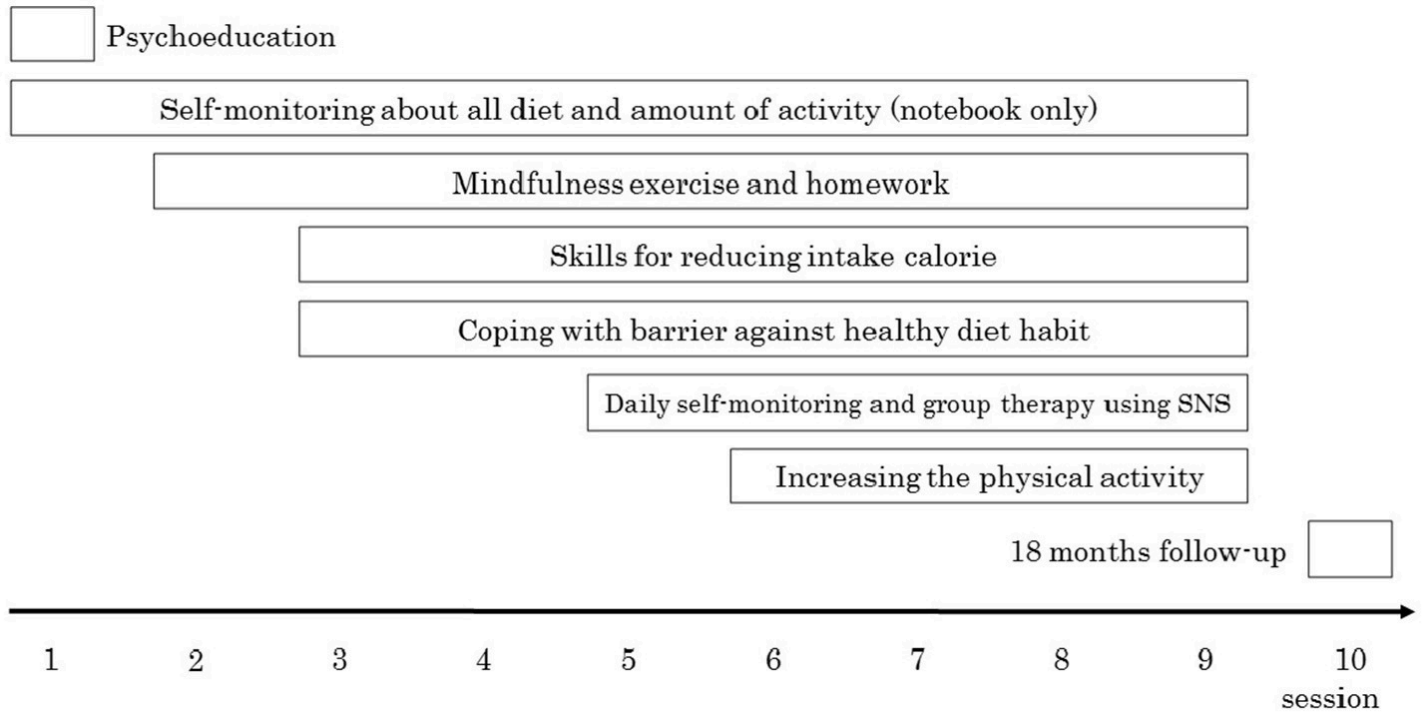
Overview of the diet program in this study. Session 1–4 was only face to face session. From session 5, participants were required to upload daily food photographs to SNS page. Session, the distance from session 9–10 was months.

The diet program consisted of weekly 60-min group sessions implemented for 9 weeks. The program was divided into two sections. Specifically, during the first 4 weeks of the program, participants received only face-to-face therapy, and in the subsequent 5 weeks of the program, participants were required to upload their daily food intake and activities to an SNS page (e.g., Facebook) and discuss them with other participants in a supportive manner (Figure 1). Participants and therapists made positive comments regarding participants' adaptive eating behaviors and suggested additional ideas concerning adaptive thoughts and the implementation of action plans in critical situations involving food-related temptation, such as dinner parties. A follow-up assessment was performed 18 months later.

### Outcomes

The primary outcome was BMI, which was calculated using participants' weight and height, and secondary outcomes were scores on the Dutch Eating Behavior Questionnaire [DEBQ; (16, 17)]. State-Trait Anxiety Inventory [STAI; (13, 14)], and Five Facet Mindfulness Questionnaire [FFMQ; (18, 19)].

The DEBQ is a 33-item self-report questionnaire that assesses comprehensive eating behavior associated with obesity. It contains subscales for three different types of eating behaviors: restrained, emotional, and external eating. Total scores on each subscale are derived from average item responses, with higher scores reflecting greater emotional and external eating, and lower scores reflecting more restrained eating.

The STAI is a 40-item self-report instrument that is widely used for the assessment of state and trait anxiety. State anxiety is transitory in nature and is characterized by subjective feelings of tension, apprehension, and nervousness. Trait anxiety refers to relatively stable individual differences in anxiety proneness, with higher scores indicating more serious symptoms of anxiety.

The FFMQ is 39-item self-report questionnaire that measures five aspects of mindfulness: observing, describing, acting with awareness, nonjudging, and nonreactivity to inner experience. Observing means noticing or attending to internal and external experiences, such as sensations, thoughts, or emotions. Describing refers to labeling internal experiences with words. Acting with awareness includes focusing on one's activities in the moment as opposed to behaving mechanically. Nonjudging refers to taking a non-evaluative stance toward thoughts and feelings. Nonreactivity to inner experience refers to allowing thoughts and feelings to come and go, without being caught up in or carried away by them. Each item is rated on a 5-point Likert scale (1 = Never or rarely true; 5 = Very often or always true), with higher scores reflecting greater mindfulness.

BMI was assessed at every session, and the DEBQ, STAI, and FFMQ were administered at baseline (Session 1), Session 9, and the follow-up assessment 18 months after Session 9.

### Ethics

The study was approved by the University of Kagoshima Human Research Ethics Committee (registered No. 26-13). Written informed consent was obtained from all participants before participation.

### Result

**Figure 2:**
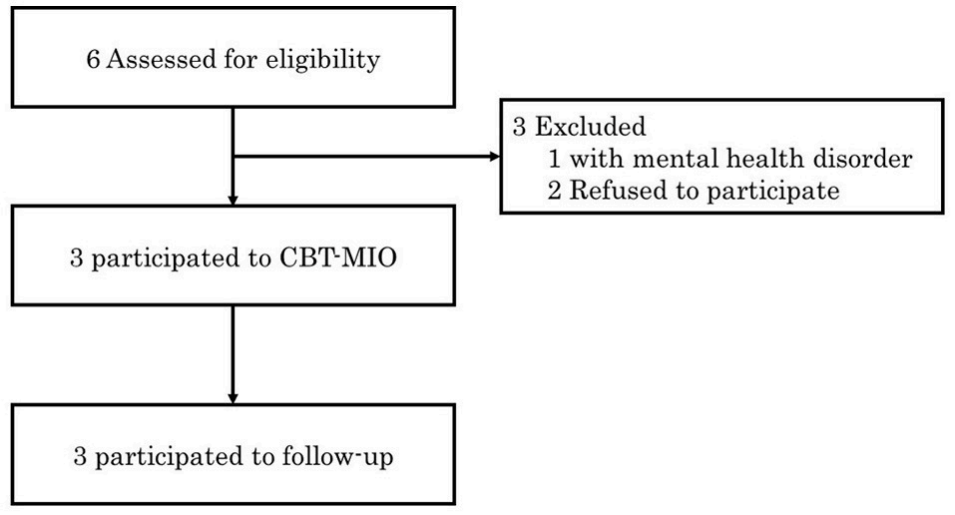
Flow of participants through the pilot study.

Figure 2 shows the participants flow through this case series. All participants succeeded in losing more than 5% of their total body weight. The proportions of total body weight lost by individual participants were 6.91% (Participant A), 5.30% (Participant B), and 8.88% (Participant C). As shown in Table 2, all participants also exhibited additional weight loss at the follow-up assessment (Participant A: 13.98%, Participant B: 7.91%, Participant C: 10.98%). Moreover, relative to baseline, participants' scores on the DEBQ restrained subscale and the FFMQ increased, whereas their scores on the DEBQ emotional and external subscales and the STAI decreased (Table 2).

**Table 2:** Changes to weight and psychological health before, during, and after treatment.

	**Participant A**			**Participant B**			**Participant C**		
	**Baseline**	**Initial weight loss (%)**	**Weight loss at follow-up (%)**	**Baseline**	**Initial weight loss (%)**	**Weight loss at follow-up (%)**	**Baseline**	**Initial weight loss (%)**	**Weight loss at follow-up (%)**
BMI (kg/m^2^)	25.31	23.56 6.91%	21.77 13.98%	29.65	28.08 5.30%	27.09 7.91%	25.22	22.98 8.88%	22.46 10.98%
**STAI SCORES**									
State	37	22	20	34	32	24	31	39	31
Trait	39	28	20	46	39	30	33	36	32
**DEBQ SCORES**									
Restrained	3.10	4.00	4.00	1.41	4.30	3.00	3.80	3.50	4.60
Emotional	3.69	2.23	2.00	1.31	1.15	1.00	1.54	1.08	1.00
External	4.10	3.00	3.70	4.10	2.50	2.00	2.30	2.10	2.30
**FFMQ SCORES**									
Observing	26	37	37	18	20	21	25	23	30
Describing	31	32	33	24	26	27	24	27	30
Acting with awareness	28	29	36	29	29	29	27	31	34
Nonjudging	21	21	32	27	35	32	30	37	29
Nonreactivity	18	26	30	19	26	24	23	23	31

*BMI, Body Mass Index; DEBQ, Dutch Eating Behavior Questionnaire; FFMQ, Five Facet Mindfulness Questionnaire; STAI, State-Trait Anxiety Inventory*.

*Weight loss was calculated as the proportion of total body weight lost*.

*Baseline = Session 1, Initial weight loss = Session 9. Weight loss at follow-up = 18 months after Session 9*.

## Discussion

The purpose of this case series was to examine the effectiveness of CBT-MIO in previously unsuccessful overweight participants, which was designed to promote weight loss and prevent dropout and subsequent weight gain. With respect to dropout, all of the participants completed the program, including the follow-up assessment, suggesting that CBT-MIO was effective in preventing dropout.

With regard to weight reduction, all participants succeeded in losing more than 5% of their total body weight. Considering that the weight loss goals for obese and overweight individuals in previous studies using CBT are between 5 and 10% (2, 3, 20, 21), the rate of weight reduction in the current study was satisfactory. In addition, while rebound weight gain is commonly observed in the first year following initial weight loss, all of the participants in the current study achieved further weight loss during the 18-month follow-up period (22).

Furthermore, all of the participants demonstrated positive changes in psychological health, and these effects persisted at the 18-month follow-up assessment. These positive changes in psychological health may have resulted from the mindfulness exercise, as previous research has also found that mindfulness interventions reduce STAI scores and increase FFMQ scores (23, 24). In view of these findings, the mindfulness exercises designed to promote appropriate eating behavior in CBT-MIO were effective in improving participants' psychological health, as reflected by changes in their DEBQ, STAI, and FFMQ scores. These results indicate that simply noticing bodily sensations, thoughts, memories, emotions, and fantasies via mindfulness exercises may be a particularly salient feature in changing conditioned patterns of eating (25). Indeed, during CBT-MIO sessions in the current study, participants reported that they recognized their impulse to eat and their automatic eating behaviors (e.g., binge eating, picking, and stress-related eating) by achieving a “here and now” awareness of their behavior, thoughts, bodily sensations, and feelings. In doing so, they were able to consider alternative cognitive behavioral plans for eating. Therefore, mindfulness may serve as a reminder that prompts individuals to use established cognitive behavioral strategies in managing their eating behaviors, instead of reverting to more maladaptive coping strategies.

Despite the above findings, the current study is subject to several limitations. First, the study sample was very small. In addition, the study was a single-arm, open study that did not include a control group. Moreover, participants used their smartphones to upload photographs of self-reported food consumption onto SNSs. Given that all participants in the current study were familiar with smartphones, this program would not be suitable for individuals who do not use or are not familiar with smartphones (e.g., older adults or individuals with financial problems). Future studies that include larger samples and control groups, such as randomized controlled trials, could address these limitations.

## Concluding remarks

In conclusion, CBT-MIO may be an effective method to facilitate weight reduction in obese and overweight individuals, prevent patient dropout, and promote the weight maintenance.

## Author contributions

KO and KK conducted the CBT-MIO sessions and wrote the paper. MA, HA, and AA made medical contributions to the design of CBT-MIO. AI made substantial, direct, and intellectual contributions to the work and approved it for publication.

### Conflict of interest statement

The authors declare that the research was conducted in the absence of any commercial or financial relationships that could be construed as a potential conflict of interest.

## References

[1] Fabricatore AN. Behavior therapy and cognitive-behavioral therapy of obesity: is there a difference? J Am Diet Assoc. (2007) 107:92–9. 10.1016/j.jada.2006.10.005 17197276

[2] Cooper Z Fairburn CG. A new cognitive behavioural approach to the treatment of obesity. Behav Res Ther. (2001) 39:499–511. 10.1016/S0005-7967(00)00065-6 11341247

[3] Cooper Z Doll HA Hawker DM Byrne S Bonner G Eeley E. Testing a new cognitive behavioural treatment for obesity: a randomized controlled trial with three-year follow-up. Behav Res Ther. (2010) 48:706–13. 10.1016/j.brat.2010.03.008 20691328 PMC2923743

[4] Lantz H Peltonen M Ågren L Torgerson JS. A dietary and behavioural programme for the treatment of obesity: a 4-year clinical trial and a long-term posttreatment follow-up. J Intern Med. (2003) 254:272–9. 10.1046/j.1365-2796.2003.01187.x 12930237

[5] HadŽiabdić MO Mucalo I Hrabač P Matić T Rahelić D BoŽikov V. Factors predictive of drop-out and weight loss success in weight management of obese patients. J Hum Nutr Diet. (2015) 28:24–32. 10.1111/jhn.12270 25220046

[6] Cuijpers P Donker T van Straten A Li J Andersson G. Is guided self-help as effective as face-to-face psychotherapy for depression and anxiety disorders? A systematic review and meta-analysis of comparative outcome studies. Psychol Med. (2010) 40:1943–57. 10.1017/S0033291710000772 20406528

[7] Dear BF Zou JB Ali S Lorian CN Johnston L Sheehan J. Clinical and cost-effectiveness of therapist-guided Internet-delivered cognitive behavior therapy for older adults with symptoms of anxiety: a randomized controlled trial. Behav Ther. (2015) 46:206–17. 10.1016/j.beth.2014.09.007 25645169

[8] Blom K Tarkian Tillgren H Wiklund T Danlycke E Forssén M Söderström A. Internet- vs. group-delivered cognitive behavior therapy for insomnia: a randomized controlled non-inferiority trial. Behav Res Ther. (2015) 70:47–55. 10.1016/j.brat.2015.05.002 25981329

[9] Andersson G. Using the Internet to provide cognitive behaviour therapy. Behav Res Ther. (2009) 47:175–80. 10.1016/j.brat.2009.01.010 19230862

[10] Kabat-Zinn J. An outpatient program in behavioral medicine for chronic pain patients based on the practice of mindfulness meditation: theoretical considerations and preliminary results. Gen Hosp Psychiatry (1982) 4:33–47. 10.1016/0163-8343(82)90026-3 7042457

[11] Hofmann SG Sawyer AT Witt A Oh D. The effect of mindfulness-based therapy on anxiety and depression: a meta-analytic review. J Consult Clin Psychol. (2010) 78:169–83. 10.1037/a0018555 20350028 PMC2848393

[12] Jenkins KT Tapper K. Resisting chocolate temptation using a brief mindfulness strategy. Br J Health Psychol. (2014) 19:509–22. 10.1111/bjhp.12050 23678870

[13] Spielberger CD Gorsuch RL Lushene RE Manual for the State-Trait Anxiety Inventory. Palo Alto, CA: Consulting Psychologists Press (1970).

[14] Nakazato K Mizuguchi T How to Use STAI. Kyoto: Sankyoubou Corp (1982).

[15] Kabat-Zinn J Full Catastrophe Living: How to Cope with Stress, Pain and Illness Uing Mindfulness Meditation. New York, NY: Bantam (2013).

[16] Van Strien T Breteler MHM Ouwens MA Restraint scale, its sub-scales concern for dieting and weight fluctuation. Pers Individ Dif. (2002) 33:791–802. 10.1016/S0191-8869(01)00192-1

[17] Naoko T Amemiya T Nishikara K Yoshizu J Ariyoshi H Suzaki Y Research on eating behaviors of adult workers and adolescent students using the Dutch Eating Behavior Questionnaire. Nihon Kenkou Igakukai Zasshi (J Japan Health Med Assoc) (2012) 21:87–94.

[18] Baer RA Smith GT Hopkins J Krietemeyer J Toney L. Using self-report assessment methods to explore facets of mindfulness. Assessment (2006) 13:27–45. 10.1177/1073191105283504 16443717

[19] Sugiura Y Sato A Ito Y Murakami H Development and validation of the Japanese Version of the Five Facet Mindfulness Questionnaire. Mindfulness (2012) 3:85–94. 10.1007/s12671-011-0082-1

[20] Wadden TA West DS Delahanty L Jakicic J Rejeski J Williamson D. The Look AHEAD study: a description of the lifestyle intervention and the evidence supporting it. Obesity (Silver Spring) (2006) 14:737–52. 10.1038/oby.2006.84 16855180 PMC2613279

[21] National Heart Lung and Blood Institute Clinical Guidelines on the Identification, Evaluation, and Treatment of Overweight and Obesity in Adults—The Evidence Report. National Institutes of Health, Obesity Research, 51S-209S. 9813653

[22] Sumithran P Prendergast LA Delbridge E Purcell K Shulkes A Kriketos A. Long-term persistence of hormonal adaptations to weight loss. N Engl J Med. (2011) 365:1597–604. 10.1056/NEJMoa1105816 22029981

[23] Haines J Spadaro KC Choi J Hoffman LA Blazeck AM. Reducing stress and anxiety in caregivers of lung transplant patients: benefits of mindfulness meditation. Int J Organ Transplant Med. (2014) 5:50–6. 25013679 PMC4089339

[24] Bränström R Kvillemo P Brandberg Y Moskowitz JT. Self-report mindfulness as a mediator of psychological well-being in a stress reduction intervention for cancer patients: a randomized study. Ann Behav Med. (2010) 39:151–61. 10.1007/s12160-010-9168-6 20177843

[25] Dalen J Smith BW Shelley BM Sloan AL Leahigh L Begay D. Pilot study: Mindful Eating and Living (MEAL): weight, eating behavior, and psychological outcomes associated with a mindfulness-based intervention for people with obesity. Complement Ther Med. (2010) 18:260–4. 10.1016/j.ctim.2010.09.0008 21130363

